# Draft Genome Sequence of *Anoxybacillus mongoliensis* Strain MB4, a Sulfur-Utilizing Aerobic Thermophile Isolated from a Hot Spring in Tattapani, Central India

**DOI:** 10.1128/genomeA.01709-16

**Published:** 2017-03-02

**Authors:** Parul Mittal, Rituja Saxena, Vineet K. Sharma

**Affiliations:** Metagenomics and Systems Biology Laboratory, Department of Biological Sciences, Indian Institute of Science Education and Research, Bhopal, Madhya Pradesh, India

## Abstract

*Anoxybacillus mongoliensis* strain MB4, an aerobic thermophile, was isolated from a hot spring located in central India. Its first draft genome sequence reported in this study comprises 2,807,516 bp and 2,853 protein-coding genes. Detailed genomic analysis indicates that it is capable of performing sulfur metabolism.

## GENOME ANNOUNCEMENT

The propensity of thermophiles to thrive in high temperatures and produce heat-stable enzymes is of immense importance to industrial and biotechnological processes (1). Several species of *Anoxybacillus* have been isolated from extreme environments, such as hot springs and geothermal soil (2–4). *A. mongoliensis*, a thermostable proteinase-producing bacterium, was first isolated from an alkaline hot spring in Mongolia (5). *A. mongoliensis* strain MB4 was isolated from the Tattapani hot spring in Sarguja district of Chhattisgarh, India (23.41°N latitude and 83.39°E longitude). The pH, temperature, and TDS (total dissolved solids) of this site were recorded to be 7.6, 61.5°C, and 600 ppm (parts per million), respectively. Chemolithotrophic thermophiles, such as *Gulbenkiania mobilis* and *Tepidimonas taiwanensis*, have been isolated from other hot springs in the central region of India (6, 7). A detailed metagenomic study of Tattapani hot springs has revealed several resident thermophiles and their survival pathways in the thermophilic environment (8).

MB4 was isolated under aerobic conditions on Difco Nutrient Agar medium (Becton, Dickinson and Company, USA) at 65°C. The colonies appeared within 24 to 48 h of incubation and were small, circular, opaque, and white in color with convex elevation. The bacterium was observed to be Gram-positive and rod-shaped, and a phenol-chloroform method was used for genomic DNA extraction using pure culture.

The 16S rRNA sequence of MB4 was BLAST-aligned with the NCBI database and showed 99% sequence identity with *A. mongoliensis* strain T4, indicating MB4 to be a novel strain of this species (9). The genome of *A. mongoliensis* strain MB4 was sequenced on the Illumina NextSeq500 platform (Illumina, USA), generating 150-bp paired-end reads. A total of 1.4 Gb of data were produced after sequencing was quality-filtered, and 7,463,715 reads were used in the assembly. The size of the draft genome assembled using SPAdes version 3.5.0 (10) is 2,807,516 bp in 93 contigs (length ≥500 bp), with an *N*_50_ contig length of 78,095 bp, an average length of 30,188.3 bp, and a G+C content of 58.3%. A total of 2,853 protein-coding genes were identified using Glimmer version 3.02 (11) and were further validated and functionally annotated using BLAST alignment with the NCBI-NR database. A total of 77 tRNAs and one rRNA were identified using tRNAscan-SE version 1.3.1 and RNAmmer version 1.2 (12, 13). The metabolic pathway identification was carried out using the KEGG Automated Annotation Server (KAAS) (14). The analysis revealed that the genome possesses an entire set of genes for glycolysis, tricarboxylic acid cycle, oxidative phosphorylation, and flagella formation. The strain also contains genes for nucleotide and mismatch repair and for converting sulfate to sulfide for energy generation.

### Accession number(s).

This whole-genome shotgun project has been deposited in DDBJ/ENA/GenBank under the accession number MRZM00000000. The version described in this paper is the first version, MRZM01000000.
